# Prediction of reproductive performance of ewes based on the early production data, ewe birth rank, dam age, and dam birth rank

**DOI:** 10.5194/aab-66-145-2023

**Published:** 2023-03-30

**Authors:** Ivan Vlahek, Velimir Sušić, Anamaria Ekert Kabalin, Sven Menčik, Maja Maurić Maljković, Aneta Piplica, Juraj Šavorić, Siniša Faraguna

**Affiliations:** Faculty of Veterinary Medicine, University of Zagreb, Vjekoslava Heinzela 55, 10000 Zagreb, Croatia

## Abstract

This research aimed to analyze whether ewes' total reproductive performance up to the fourth year of life (RP4) can be predicted based on the data available at an early stage of a ewe's productive life. The RP4 of 133 Romanov ewes was measured in terms of the total number of lambs born per ewe (TNLE) and total birth weight of lambs per ewe (TBLE). Multiple regression was used to analyze whether early reproductive performance indicators (first litter size – FLS, age at first lambing – AFL, first lambing interval – FLI), ewe birth rank, dam age, and dam birth rank can be used as the predictors of RP4. Predicted $R^{2}$ and 95 % prediction intervals were used as indicators of the precision of prediction. Average TNLE and TBLE at the end of fourth year of ewe life were 11.84 lambs and 37.96 kg, respectively. FLS and FLI significantly ( P<0.05 ) influenced TNLE and TBLE, while AFL was not a significant ( P>0.05 ) variable. Ewes with shorter FLI had significantly ( P<0.05 ) higher TNLE (10.94 lambs) and TBLE (36.17 kg) than ewes with long FLI (TNLE = 9.12 lambs and TBLE = 28.05 kg). $R^{2}$ predicted for TNLE and TBLE was 7.54 % and 11.49 %, respectively. The ewe's birth rank and the dam's birth rank significantly ( P<0.05 ) influenced TNLE and TBLE. Ewes born as singletons and ewes from singleton-born dams had significantly ( P<0.05 ) lower TNLE and TBLE than ewes born as triplets and ewes from triplet-born dams. $R^{2}$ predicted for TNLE was 16.76 %, and 25.69 % for TBLE. FLS and FLI are better predictors of RP4 than AFL. The birth rank of ewe and dam also proved significant predictors of RP4. For both sets of predictors (early reproductive indicators and birth rank data), low values of $R^{2}$ predicted indicate that precise prediction of RP4 cannot be made.

## 1 Introduction

The reproductive performance of sheep is one of the main factors on which the efficiency of meat production systems depends. Frequently used indicators of the reproductive performance of sheep include fertility, prolificacy, sexual maturity, age at first lambing, lambing interval, conception rates, and lambs weaned/ewe/year (Vanimisetti and Notter, 2012; Assan, 2020). Total reproductive performance measured in terms of the total number of lambs born per ewe over a long period or in a lifetime is used less frequently but is one of the best indicators of overall sheep productivity and value (Lee et al., 2014).

In annual lambing systems, total reproductive performance depends on the component traits such as fertility, fecundity, and lamb survival. In the accelerated lambing systems, ewes may lamb more than once per year, making the lambing frequency (lambing interval) an essential component trait (Vanimisetti and Notter, 2012).

Performance records are often used as one of the primary sources of information for selecting replacements and culling ewes (Santos et al., 2017). Total reproductive performance usually contributes little to the selection or culling decisions due to late availability. However, if it could be predicted using the reproductive indicators recorded earlier in the sheep's life, total reproductive performance could be an important factor in making that decision. In predictive modeling, the imperative is to produce precise and unbiased predictions, and predictive models usually require inexpensive and measurable data (Frost, 2019). In that sense, early reproductive performance indicators (e.g., first litter size, age at first lambing, first lambing interval) are promising candidate predictors for the total reproductive performance of ewes. Also, dam age and ewe birth rank, as investigated by Pettigrew et al. (2019), might have a significant influence on ewe total reproductive performance and thus be used as its predictors.

Generating the prediction values of reproductive traits in sheep can be performed using various statistical procedures, including a regression approach (Schoeman et al., 1991; Lee and Atkins, 1996) and data mining algorithms such as neural networks and decision trees (Zaborski et al., 2019). So far, the regression approach was more common in determining the favorable predictors of the lifetime performance of ewes. However, the precision of prediction based on the $R^{2}$ predicted and prediction intervals was not analyzed yet.

This research aimed to assess the influence and predictive value of several ewe early reproductive performance indicators, ewe birth rank, dam age, and dam birth rank on the total reproductive performance of sheep up to the fourth year of life (RP4).

## 2 Material and methods

### 2.1 Location, animals, and general management

The study was conducted between 2015 and 2020 at one commercial semi-intensive sheep farm in Croatia located at 45 ${}^{\circ }$ 22 ${}^{\prime }$ N, 14 ${}^{\circ }$ 38 ${}^{\prime }$ E. Overall reproductive management was based on accelerated lambing with continuous mating. Romanov ewe lambs born from February to April (5–7 months of age) were randomly allocated into four breeding groups, each containing 35–40 ewe lambs and one ram. Joining at 5–7 months of age was possible because Romanov sheep is early maturing breed, and all ewe lambs were well fed and thus attained at least 60 % of their mature weight. The rams were 1 year old, healthy, reproductively viable, and unrelated to the ewes. Lambing occurred indoors, with every pregnant ewe giving birth in a small separate pen. Three to four days after lambing, ewes and lambs were returned to the ewe's breeding groups. The weaning of lambs occurred at 45 d of age. Grazing was limited due to small outdoor pens, and all animals were fed a commercial feed mixture with 16 % protein and meadow hay ad libitum.

### 2.2 Measurements and data processing

Initially, a total of 148 ewes were included in the study. Excluding criteria were missing data, age at first lambing longer than 600 d, and lambing interval longer than 500 d. At the end of the study period, 15 ewes were excluded from the analyses. Measurements ended after ewe reached 4 years of life. A 4-year threshold was set because most of the Romanov ewes reached and passed their maximum productivity during this time (Fahmy, 1996). Records on ewes included their birth rank (size of the litter in which ewe was born), date of birth, date of first and second lambings, litter sizes, and birth weight of lambs. Dam birth rank (size of the litter in which dam was born) and dam age (ewe lamb or mature ewe) were also collected. A young female dam giving birth for the first time was considered a ewe lamb, while mature ewes (dams) lambed two or more times. Lambs were weighed within 12 h after birth. Age at first lambing and first lambing interval were calculated using the obtained records. Based on the age at first lambing, ewes were divided into groups as follows: early ( <350 d), medium (350–400 d), and late ( >400 d). Lambing interval groups were determined as follows: short ( <235 d), medium (235–285 d), and long ( >285 d).

Reproductive performance was measured in terms of the total number of lambs born per ewe and the total birth weight of lambs per ewe. For the analyses, four variables were created. A total number of lambs per ewe 1 (TNLE1) was calculated as the total number of live-born lambs except first lambing, and the total birth weight of lambs per ewe (TBLE1) as the sum of all birth weights of lambs, except those from first lambing. The total number of lambs per ewe 2 (TNLE2) was determined as the total number of live-born lambs per ewe and the total birth weight of lambs per ewe 2 (TBLE2) by adding up all birth weights of lambs.

### 2.3 Statistical analysis

Statistical analysis was performed in the Minitab statistical program (Minitab LLC, 2021). Two separate multivariate regression models were built. In the first model, TNLE1 and TBLE1 were evaluated based on the age at first lambing class, first litter size, and first lambing interval class as fixed factors. The equation of the model was as follows:

$$Y_{ijk}=\mu +A_{i}+{\mathrm{LS}}_{j}+{\mathrm{LI}}_{k}+e_{ijk},$$

where $Y_{ijk}$ is phenotypic value of TNLE1 and TBLE1; μ is the overall population mean; $A_{i}$ is the fixed effect of the i th age at first lambing class; LS ${}_{j}$ is the fixed effect of the j th litter size; LI ${}_{k}$ is fixed effect of the k th lambing interval class; $e_{ijk}$ is residual error.

The second model was built to analyze TNLE2 and TBLE2 and included the age of the dam, dam birth rank, and ewes' birth rank as fixed factors. The model written in mathematical form was as follows:

$$Y_{ijk}=\mu +{\mathrm{AD}}_{i}+{\mathrm{LD}}_{j}+{\mathrm{LE}}_{k}+e_{ijk},$$

where $Y_{ijk}$ is the phenotypic value of TNLE2 and TBLE2; μ is the overall population mean; AD ${}_{i}$ is the fixed effect of the i th age of dam; LD ${}_{j}$ is the fixed effect of the j th dam birth rank; LE ${}_{k}$ is the fixed effect of the k th birth rank of ewe; $e_{ijk}$ is the residual error.

ANOVA procedure was used to test for the significant effect of fixed factors, and the Tukey post hoc test for an unequal number of samples was used to test the significance of differences between groups within the same fixed factor. The significance level was set at P<0.05 .

Akaike information criterion (AIC) was used to determine the best model for the prediction of RP4. After the formation of the optimal models, predicted $R^{2}$ and a 95 % prediction interval were determined. Predicted $R^{2}$ is used to assess the model's goodness of fit for the prediction of independent variable values (it indicates how well the model fits the observed data). The 95 % prediction interval is the range where a single new observation is 95 % likely to fall, given the specific values of the independent variables (Frost, 2019).

## 3 Results

### 3.1 Descriptive statistics

During the 4-year period investigated, ewes produced, on average, 11.84 lambs, with an average total lamb weight of 37.96 kg (Table 1). The coefficient of variation suggests relative homogeneity of the data.

**Table 1:** Descriptive statistics for the total number of lambs per ewe 1 and total birth weight of lambs per ewe 1.

Variable	Descriptive parameter					
	n	Mean	SD	Min	Max	CV %
Total number of lambs per ewe 1	133	11.84	2.33	7.00	18.00	19.64
Total birth weight of lambs per ewe 1 (kg)	133	37.96	7.55	21.48	57.01	19.90

n – number of ewes; SD – standard deviation; Min – minimum; Max – maximum; CV % – coefficient of variation %.

### 3.2 Inferential statistics

The proportion of variation explained by the model with early reproductive performance indicators was 15.4 % ( $R^{2}=0.154$ , F(6,126) = 3.81, P=0.002 ) for TNLE1 and 18.6 % ( $R^{2}=0.186$ , F(6,126) = 4.80, P<0.001 ) for TBLE1. ANOVA procedure showed that the TNLE1 was significantly affected by litter size and lambing interval, while the lambing interval was significant ( P<0.05 ) in the model for TBLE1. Ewes with short lambing intervals had significantly ( P<0.05 ) higher TBLE1 compared to the ewes with long lambing intervals (Table 2). The birth rank of the ewe, the birth rank of the dam, and the age of the dam explained 21.2 % ( $R^{2}=0.212$ , F(5,125) = 6.74, P<0.001 ) and 33.8 % ( $R^{2}=0.386$ , F(5,125) = 12.74, P<0.001 ) variation in TNLE2 and TBLE2, respectively. In both models, the ewe's birth rank and the dam's birth rank were significant ( P<0.05 ) variables. Ewes born as singletons and ewes from singleton-born dams had significantly ( P<0.05 ) lower TNLE2 and TBLE2 than ewes born as triplets and ewes from triplet-born dams (Table 3).

**Table 2:** Analysis of variance results of the total number of lambs per ewe 1 and total birth weight of lambs per ewe 1.

Fixed factor	Level of factor	n	Variable	
			Total number of	Total birth weight
			lambs per ewe 1	of lambs per ewe 1
			LSM ± SE	LSM ± SE
Age at first lambing	Early	36	10.36 ± 0.37	31.96 ± 1.23
	Medium	60	10.02 ± 0.32	32.56 ± 1.06
	Late	37	9.79 ± 0.43	31.20 ± 1.43
Litter size	1	21	9.46 ± 0.46	31.24 ± 1.53
	2	94	9.67 ± 0.23	31.18 ± 0.76
	3	18	11.04 ± 0.52	33.29 ± 1.72
Lambing interval	Short	43	10.94 ${}^{a}$ ± 0.39	36.17 ${}^{a}$ ± 1.31
	Medium	54	10.11 ${}^{\mathrm{ab}}$ ± 0.34	31.50 ${}^{b}$ ± 1.11
	Long	36	9.12 ${}^{b}$ ± 0.40	28.05 ${}^{b}$ ± 1.32

LSM – least square means; SE – standard error; ${}^{\mathrm{ab}}$ – least square means within the same column with different superscript letters differ statistically at the level P<0.05 .

**Table 3:** Analysis of variance results of the total number of lambs per ewe 2 and total birth weight of lambs per ewe 2.

Fixed factor	Level of factor	n	Variable	
			Total number of	Total birth weight
			lambs per ewe 2	of lambs per ewe 2
			LSM ± SE	LSM ± SE
Ewe birth rank	1	32	11.02 ${}^{a}$ ± 0.40	34.22 ${}^{a}$ ± 1.19
	2	85	12.10 ${}^{\mathrm{ab}}$ ± 0.25	38.82 ${}^{b}$ ± 0.74
	3	14	13.32 ${}^{b}$ ± 0.57	44.05 ${}^{b}$ ± 1.70
Dam birth rank	1	42	11.05 ${}^{a}$ ± 0.36	34.70 ${}^{a}$ ± 1.07
	2	68	12.17 ${}^{b}$ ± 0,30	39.55 ${}^{b}$ ± 0.90
	3	21	13.22 ${}^{b}$ ± 0.49	42.83 ${}^{b}$ ± 1.46
Age of dam	Ewe lamb	58	11.86 ± 0.34	37.67 ± 1.01
	Ewe	73	12.43 ± 0.30	40.39 ± 0.89

LSM – least square means; SE – standard error; ${}^{\mathrm{ab}}$ – least square means within the same column with different superscript letters differ statistically at the level P<0.05 .

Means and prediction intervals of TNLE1 and TBLE1 for the combination of ewe first litter size and first lambing interval are presented in Table 4. Larger litter sizes and shorter lambing intervals suggest higher TNLE1 and TBLE1, with wide prediction intervals for all combinations. $R^{2}$ predicted (indicating the precision of prediction) was 7.54 % and 11.49 % for TNLE1 and TBLE1, respectively. Ewes born as twins or triplets from triplet-born dams had the highest TNLE2 and TBLE2 (Table 5). Prediction intervals were wide, regardless of the combination of ewe and dam birth rank. $R^{2}$ predicted for TNLE2 was 16.76 %, and 25.69 % for TBLE2.

**Table 4:** Means and prediction intervals of reproductive performance up to the fourth year of ewe's life for the combination of first litter size and first lambing interval.

Combination of ewe first litter size and first lambing interval	Variable					
	Total number of lambs per ewe 1			Total birth weight of lambs per ewe 1		
	Observed	Predicted	PI 95 %	Observed	Predicted	PI 95 %
	mean	mean		mean	mean	
Singleton – short	10.83	10.47	6.28–14.65	36.22	35.70	21.85–49.55
Singleton – medium	8.75	9.45	5.23–13.68	30.86	30.42	14.48–44.37
Singleton – long	8.20	8.50	4.27–12.73	25.97	27.57	13.60–41.53
Twin – short	10.43	10.64	6.50–14.78	35.35	35.81	22.14–49.48
Twin – medium	9.79	9.62	5.49–13.75	31.00	30.53	16.90–44.16
Twin – long	8.64	8.67	4.52–12.81	27.50	27.68	13.99–41.37
Triplet – short	14,00	12.12	7.85–16.38	45.62	38.08	24.01–52.16
Triplet – medium	10.73	11.09	6.89–15.30	30.99	32.81	18.91–46.70
Triplet – long	10.50	10.14	5.91–14.37	32.05	29.95	15.97–43.93

PI 95 % – 95 % prediction interval.

**Table 5:** Means and prediction intervals of reproductive performance up to the fourth year of ewe's life for the combination of ewe birth rank and dam birth rank.

Combination of ewe birth rank and dam birth rank	Variable					
	Total number of lambs per ewe 2			Total birth weight of lambs per ewe 2		
	Observed	Predicted	PI 95 %	Observed	Predicted	PI 95 %
	mean	mean		mean	mean	
Singleton – singleton	10.60	10.33	6.15–14.51	32.65	31.15	18.29–44.00
Singleton – twin	10.73	11.22	7.05–15.40	34.03	35.83	22.99–48.67
Singleton – triplet	12.67	13.31	9.06–17.56	37.51	41.78	28.72–54.85
Twin – singleton	11.00	11.07	6.90–15.23	33.43	34.65	21.85–47.46
Twin – twin	12.00	11.97	7.83–16.10	39.59	39.34	26.61–52.07
Twin – triplet	13.53	14.05	9.86–18.25	43.78	45.29	32.39–58.18
Triplet – singleton	11.00	11.06	6.70–15.43	40.32	35.03	21.61–48.45
Triplet – twin	15.00	11.96	7.62–16.29	46.28	39.71	26.38–53.05
Triplet – triplet	12.33	14.05	9.66–18.43	43.91	45.66	32.18–59.15

PI 95 % – 95 % prediction interval.

## 4 Discussion

Reproductive indicators measured earlier in life might be helpful in making a selection and culling decisions, given that they are, to some extent, associated with the ewes' total or lifetime reproductive performance. Authors of several studies used this presumption in an effort to explain or predict ewe reproductive performance (Lee and Atkins, 1996; Amer et al., 2007; Lee et al., 2014; Kleemann et al., 2016) or its stayability (Douhard et al., 2016).

In our research, the total number of lambs per ewe and the total birth weight of lambs per ewe were analyzed. Early reproductive performance indicators (first litter size and first lambing interval) were significantly associated with ewe RP4. This result was expected given that the litter size is the indicator most closely connected to the total reproductive performance. Indeed, Lee and Atkins (1996) found that, in the commercial Merino flock, early life fecundity was indicative of fertility, fecundity, and the rearing ability of ewes in later life. Based on the results from our research, ewes with short first lambing intervals had significantly higher production in terms of the number of lambs and birth weight of lambs than ewes whose first lambing interval was long. This may be because some of the variations in lambing intervals are repeatable, so ewes with short first lambing intervals lambed more frequently than ewes with long first lambing interval. Areb et al. (2021) found the repeatability of the lambing interval in Bonga sheep to be as high as 0.57. They concluded that selection decisions could be made based on the early reproductive performance indicators. However, this suggestion should be the topic of further and more extensive research, given that the repeatability of lambing intervals is usually below 0.10 (Iniguez et al., 1991; Schoeman et al., 1991; María, 1995; Monforte et al., 2013; Canché et al., 2016).

Although all ewes in our research were initially joined with rams at the same time, the age at first lambing varied by 3 months. However, differences in RP4 were not significant, meaning that the Romanov ewes in the continuous mating system can be mated at a relatively large age span without hindering their reproductive efficiency in later life. This seems like valuable information, given that the age at first mating/lambing is considered one of the main determinants of ewes' productive life (Kenyon et al., 2011; Foster and Hileman, 2015) and has a negative genetic correlation with lifetime reproduction (Jafaroghli et al., 2022). Indeed, it was shown that age at first lambing affects stayability (Mclaren et al., 2020) and lifetime reproductive performance of ewes in pelt (Schoeman et al., 1991), dairy (Hernandez et al., 2011), and meat (Thomson et al., 2021) production systems. It should be pointed out that although continuous mating provides simple reproductive management, it also has several disadvantages (the exact time of mating for an individual ewe is not known and can only be calculated after lambing; compact lambing periods do not exist and lambings occur throughout the year).

Several authors (Schoeman et al., 1991; Lee and Atkins, 1996) suggested using early reproductive performance indicators to predict the total reproductive performance of ewes. Based on the results obtained in our research, first litter size and first lambing interval seem to be likely predictors of RP4, but a precise prediction was not possible. Even though observed and predicted means were very similar in most litter size-lambing interval combination groups, the low value of the $R^{2}$ predicted and a wide range of prediction intervals indicate an imprecise estimate. However, the practical importance of results might be in determining the lower threshold of the 95 % prediction interval. For example, ewes with short first lambing intervals, which lambed triplets in the first litter, are expected to produce at least 7.85 lambs in the first 4 years of life.

The second model demonstrated that dam and ewe birth rank significantly affect the RP4. If ewes and their dams were from multiple litters, ewes' RP4 tended to be higher in terms of lamb production and birth weight. Age of the dam did not significantly influence the RP4. Reports on the influence of dam birth rank, ewe birth rank, and age of dam on the reproductive performance of ewe are scarce. Loureiro et al. (2012) investigated whether being born to a ewe lamb or adult dam affected ewes' live weight and reproductive performance of ewes. Authors found that ewes born to ewe lamb dams were significantly lighter at birth and 12 months of age than ewes born to adult dams, but no significant difference regarding reproductive performance was observed. In a recent experiment, Pettigrew et al. (2019) investigated the combined effect of the ewe's birth rank and the dam's age on several lifetime reproductive performance indicators of ewes. None of the effects was significant for the number of lambs born. However, ewes born to mature dams had significantly lighter litters at birth, and twin-born ewes born to ewe lamb dams had lighter litters than single-born ewes. Higher RP4 of multiple litter-born ewes from multiple litter-born dams might be partially attributed to the genetic variation in reproductive potential among ewes (Fahmy, 1996; Notter, 2012). However, further research on the genetic structure of reproductive traits, including the candidate genes approach, is necessary to draw valid conclusions.

$R^{2}$ predicted in the second model was low, indicating a relatively imprecise estimate, evident from the prediction intervals. Similarly, as in the first prediction model, the information about the lowest predicted performance of ewes belonging to a particular group might be helpful to breeders. This is more important insofar as the predicted total number of lambs born per ewe 1 and total birth weight of lambs per ewe 1 were more than three lambs and 14 kg higher in triplet-born ewes from triplet-born dams compared to ewes born as singletons by singleton-born dams. Thus, the multiple-born female lambs from multiple-born dams should be favored when selecting replacements.

The main limitation of this study was the relatively small sample size. However, intensive and semi-intensive sheep farming in Croatia is mainly organized in small farms containing from 100 to 200 reproductive sheep, and our intention was to investigate sheep on one farm and under the same management practices in order to minimize environmental variations. A study on the larger number of sheep might produce more reliable predictions.

## 5 Conclusion

This study confirmed the association between early reproductive performance indicators (first litter size and first lambing interval), the birth rank of ewe and birth rank of dam, and total reproductive performance measured in terms of the total number of lambs born per ewe and total birth weight of lambs per ewe. However, these indicators were of limited value as predictors of ewe total reproductive performance, mainly because they generated imprecise estimates. Including additional predictors and increasing the sample size might improve the precision of the prediction.

## Data Availability

The data used and analyzed in this study are available from the corresponding author upon reasonable request.

## References

[bib1.bib1] Amer PR, Jopson NB, Cocks J, Scarlet AL (2007). Impact of early age litter size on subsequent litter output in ewes.

[bib1.bib2] Areb E, Getachew T, Kirmani MA, Silase TG, Haile A (2021). Estimation of co-variance components, genetic parameters, and genetic trends of reproductive traits in community-based breeding program of Bonga sheep in Ethiopia. Anim Biosci.

[bib1.bib3] Assan N (2020). Indicators of reproductive performance in goats and sheep meat production. Sci J Anim Sci.

[bib1.bib4] Canché JET, Monforte JGM, Correa JCS (2016). Environmental effects on productive and reproductive performance of Pelibuey ewes in Southeastern Mexico. J Appl Anim Res.

[bib1.bib5] Douhard F, Jopson NB, Friggens NC, Amer PR (2016). Effects of the level of early productivity on the lifespan of ewes in contrasting flock environments. Animal.

[bib1.bib6] Fahmy M, Fahmy M (1996). Prolific sheep.

[bib1.bib7] Foster DL, Hileman SM, Plant TM, Zeleznik AJ (2015). Knobil and Neill's physiology of reproduction: volume two.

[bib1.bib8] Frost J (2019). Regression analysis: An intuitive guide for using and interpreting linear models.

[bib1.bib9] Hernandez F, Elvira L, Gonzales-Martin JV, Gonzales-Bulnes A, Astiz S (2011). Influence of age at first lambing on reproductive and productive performance of Lacaune dairy sheep under an intensive management system. J Dairy Res.

[bib1.bib10] Iniguez L, Sanchez M, Ginting S (1991). Productivity of Sumatran sheep in a system integrated with rubber plantation. Small Ruminant Res.

[bib1.bib11] Jafaroghli M, Ghafouri-Kesbi F, Khorami SJ, Barazandeh A, Mokhtari M (2022). Application of structural equation models for genetic evaluation of lifetime reproductive traits and age at first lambing in Moghani sheep. Small Ruminant Res.

[bib1.bib12] Kenyon PR, Van Der Linden DS, West DM, Morris ST (2011). The effect of breeding hoggets on lifetime performance. New Zeal J Agr Res.

[bib1.bib13] Kleemann DO, Walker SK, Ponzoni RW, Gifford DR, Walkley JRW, Smith DH, Grimson RJ, Jaensch KS, Walkom SF, Brien FD (2016). Effect of previous reproductive performance on current reproductive rate in South Australian Merino ewes. Anim Prod Sci.

[bib1.bib14] Lee GJ, Atkins KD (1996). Prediction of lifetime reproductive performance of Australian merino ewes from reproductive performance in early life. Anim Prod Sci.

[bib1.bib15] Lee GJ, Sladek MA, Hatcher S, Richards JS (2014). Using partial records to identify productive older ewes to retain in the breeding flock to increase flock net reproduction rate. Anim Prod Sci.

[bib1.bib16] Loureiro MFP, Pain SJ, Kenyon PR, Peterson SW, Blair HT (2012). Single female offspring born to primiparous ewe-lambs are lighter than those born to adult multiparous ewes but their reproduction and milk production are unaffected. Anim Prod Sci.

[bib1.bib17] Monforte JGM, Cab MH, López RJA, Correa JCS (2013). A field study of reproductive performance and productivity of Pelibuey ewes in southeastern Mexico. Trop Anim Health Pro.

[bib1.bib18] María GA (1995). Estimates of variances due to direct and maternal effects for reproductive traits of Romanov sheep. Small Ruminant Res.

[bib1.bib19] Mclaren A, Mchugh N, Lambe NR, Pabiou T, Wall E, Boman IA (2020). Factors affecting ewe longevity on sheep farms in three European countries. Small Ruminant Res.

[bib1.bib20] (2021). Minitab [Internet].

[bib1.bib21] Notter DR (2012). Genetic improvement of reproductive efficiency of sheep and goats. Anim Reprod Sci.

[bib1.bib22] Pettigrew EJ, Hickson RE, Morris ST, Lopez-Villalobos N, Pain SJ, Kenyon PR, Blair HT (2019). The effects of birth rank (single or twin) and dam age on the lifetime productive performance of female dual purpose sheep (*Ovis aries*) offspring in New Zealand. Plos One.

[bib1.bib23] Santos BFS, van der Werf JHJ, Gibson JP, Byrne TJ, Amer PR (2017). Genetic and economic benefits of selection based on performance recording and genotyping in lower tiers of multi-tiered sheep breeding schemes. Genet Sel Evol.

[bib1.bib24] Schoeman SJ, Albertyn JR, Groenveld HT (1991). Lifetime reproduction of Karakul ewes as influenced by season of birth, age at first lambing and lambing interval. S Afr J Anim Sci.

[bib1.bib25] Thomson BC, Smith NB, Muir PD (2021). Effect of birth rank and age at first lambing on lifetime performance and ewe efficiency. New Zeal J Agr Res.

[bib1.bib26] Vanimisetti HB, Notter DR (2012). Opportunities for genetic evaluation of reproductive performance in accelerated lambing systems. Livest Sci.

[bib1.bib27] Zaborski D, Ali M, Eyduran E, Grzesiak W, Tariq MM, Abbas F, Waheed A, Tirink C (2019). Prediction of selected reproductive traits of indigenous Harnai sheep under farm management system via various data mining algorithms. Pak J Zool.

